# Identifying developmental QTL alleles with favorable effect on grain yield components under late‐terminal drought in spring barley MAGIC population

**DOI:** 10.1002/pld3.516

**Published:** 2023-08-02

**Authors:** Nazanin P. Afsharyan, Wiebke Sannemann, Agim Ballvora, Jens Léon

**Affiliations:** ^1^ Institute for Crop Science and Resource Conservation, Chair of Plant Breeding University of Bonn Bonn Germany; ^2^ Department of Plant Breeding Justus Liebig University Giessen Giessen Germany; ^3^ KWS Saat SE & Co. KGaA Einbeck Germany

**Keywords:** barley, late‐terminal drought tolerance, MAGIC population, maker by treatment interaction, QTL analysis, yield‐related traits

## Abstract

Barley is the fourth most cultivated cereal worldwide, and drought is a major cause of its yield loss by negatively affecting its development. Hence, better understanding developmental mechanisms that control complex polygenic yield‐related traits under drought is essential to uncover favorable yield regulators. This study evaluated seven above‐ground yield‐related traits under well‐watered (WW) and late‐terminal drought (TD) treatment using 534 spring barley multiparent advanced generation intercross double haploid (DH) lines. The analysis of quantitative trait loci (QTL) for WW, TD, marker by treatment interaction, and drought stress tolerance identified 69, 64, 25, and 25 loci, respectively, for seven traits from which 15 loci were common for at least three traits and 17 were shared by TD and drought stress tolerance. Evaluation of allelic effects for a QTL revealed varying effect of parental alleles. Results showed prominent QTL located on major flowering time gene *Ppd‐H1* with favorable effects for grain weight under TD when flowering time was not significantly affected, suggesting that this gene might be linked with increasing grain weight by ways other than timing of flowering under late‐terminal drought stress. Furthermore, a desirable novel QTL allele was identified on chromosome 5H for grain number under TD nearby sucrose transporter gene *HvSUT2*. The findings indicated that spring barley multiparent advanced generation intercross population can provide insights to improve yield under complex condition of drought.

## 1 INTRODUCTION

Barley (*Hordeum vulgare* L.) is the fourth most cultivated cereal worldwide. Its domestication dates back to 10,000 years ago in arid and semiarid areas in western Asia known as Fertile Crescent (Harlan & Zohary, 1966; Wang et al., 2015). Compared with other small grain cereals, barley is known to be more tolerant to drought and harsh climate. This feature, combined with self‐pollination and diploidy, makes barley a model for highly complex polygenic traits such as yield or drought tolerance (Schulte et al., 2009; Ullrich, 2010) which are influenced by various genetic pathways and environmental factors (Ozturk et al., 2018; Sallam et al., 2019). Drought, in particular, is one of the important causes of barley yield loss (Jamieson et al., 1995; Rollins et al., 2013) as it negatively affects plant development and yield components such as number of grains per m^−2^ and thousand grain weight (Pennisi, 2008). Drought tolerance is controlled by various complex mechanisms, and therefore, improving it is a challenging task which requires better understanding plant development under drought stress (Sallam et al., 2019).

Drought can cause yield loss during all phases of plant life cycle (Salekdeh et al., 2009). However, sensitivity to drought stress varies in different stages of crop development. Early‐season drought can damage yield by reducing seedling survival during the vegetative development (Lelièvre, 1981). On the other hand, late‐season drought which occurs during reproductive development can have devastating effect on grain number per unit area and thousand grain weight and is more likely to occur in field; it can be referred to as terminal drought or late‐terminal drought (TD) (Farooq et al., 2014; Jamieson et al., 1995; Samarah et al., 2009; Samarah & Alqudah, 2011; Shavrukov et al., 2017). Loss of grain yield components including grain weight, grain number, and ear number is an indication of yield reduction in barley. However, because number of grains per m^−2^ is the most important component of cereal yield (Reynolds et al., 2009; Slafer, 2003; Sreenivasulu & Schnurbusch, 2012), reduction of ear number and grain number per ear is reported to be strongly connected to yield loss under drought stress (Samarah et al., 2009). Drought stress can reduce number of grains per m^−2^ by causing loss of spikelets, increasing floret sterility, and loss of seed set (Dolferus et al., 2011; Svobodová & Míša, 2004). After anthesis and seed set, the main negative effect of drought is by reducing the grain filling rate and duration by restricting the final number of endosperm cells or limiting the rate and duration of starch accumulation in the endosperm (Radchuk et al., 2009; Setter & Flannigan, 2001; Wallwork et al., 1998).

Gaining more insights into role of genetic mechanisms controlling plant phenology and development and their influence on grain yield components under drought is mandatory for developing high‐yielding drought‐tolerant barley cultivars (Cattivelli et al., 2008). Among developmental events, flowering time is a key trait which is controlled by genetic mechanisms that interact with environment (Afsharyan et al., 2020). Impact of drought stress on regulation of flowering time genes can affect grain yield components (Gol et al., 2020; Wiegmann et al., 2019). Major flowering time loci were mapped in association with grain yield components under drought stress such as *Vrn‐H1* gene and *denso/sdw1* gene for tiller number and *Vrn‐H3* (*HvFT1*) gene for fresh weight (Pham et al., 2019). However, little is known about the contribution of developmental mechanisms including flowering time to yield during TD, when drought has little or no influence on timing of flowering. One study found that photoperiod gene *PSEUDO‐RESPONSE REGULATOR Ppd‐H1* (*HvPRR37*) locus is linked with plant biomass and thousand grain weight when drought treatment was implemented after flowering time (Honsdorf et al., 2017). Therefore, the contribution of development and flowering time genetic loci to yield during late‐terminal drought stress is not well elaborated yet.

To empower quantitative trait loci (QTL) detection, past QTL mapping efforts in barley used various approaches such as advanced‐backcross population (Sayed et al., 2012), introgression lines (Honsdorf et al., 2014, 2017), and association panels (Wehner et al., 2015). In addition to mapping QTL associated with traits under normal and drought conditions, QTL were also detected based on stress indices as phenotype input (Honsdorf et al., 2014; Oyiga et al., 2020). Fernandez et al. (1992) proposed stress tolerance (ST) index (STI) which is calculated based on phenotyping data from normal and stress conditions to identify genotypes that show better tolerance under stress condition. This index was used previously to directly detect QTL associated with drought tolerance (Oyiga et al., 2020). However, QTL mapping under drought condition proved complicated due to large genotype by environment interactions which resulted in detecting smaller effect QTL (Abdel‐Haleem et al., 2012; Honsdorf et al., 2014; Li et al., 2013; Wehner et al., 2015). QTL mapping for drought tolerance was also challenging due to its complex nature including contribution of many genes with small effects (Honsdorf et al., 2014; Sallam et al., 2019). Reports for using a more advanced mapping approach such as multiparental populations are rare. One study used a nested association mapping population to study traits such as dry weight, fresh weight, plant height (PLH), tiller number under control, and drought condition (Pham et al., 2019). Multiparent advanced generation intercross (MAGIC) strategy was designed to increase the power of QTL mapping (Bandillo et al., 2013; Cavanagh et al., 2008; King et al., 2012). In this study, we used a spring barley MAGIC population constructed from eight‐way cross of seven landraces known as “founders of German barley” and one elite cultivar Barke (Sannemann et al., 2015) which has been utilized recently in a series of flowering time studies. A recent study which used this MAGIC population showed very precise recognition of known flowering time loci as a proof of concept, in addition to identifying new loci (Afsharyan et al., 2020; Mathew et al., 2018; Maurer et al., 2017; Sannemann et al., 2015).

This study aims to investigate the contribution of genetic mechanisms involved in plant development and flowering time to grain yield components and tolerance under late‐terminal drought stress. For this purpose, spring barley MAGIC DH lines were used for evaluating and QTL mapping seven above‐ground yield‐related traits under well‐watered (WW) and late‐terminal drought (TD) treatment. Effects of QTL alleles associated with major flowering time and developmental traits as well as grain yield traits were investigated to detect QTL with favorable effect on grain yield components and drought tolerance.

## 2 MATERIAL AND METHODS

### 2.1 Plant material and experimental setup

**FIGURE 1:**
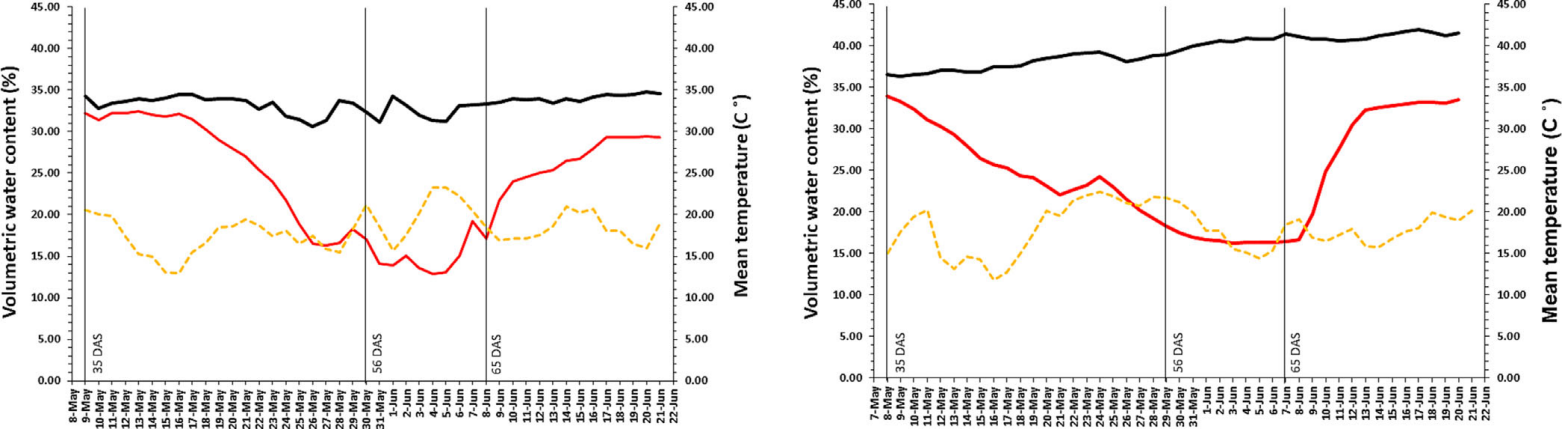
Soil moisture content for well‐watered (black) and terminal drought (red) treatments as well as daily mean temperature (orange) in 2011 and 2012. The days after sowing (DAS) in the start of reduction in irrigation, permanent wilting point, and rewatering is indicated for late‐terminal drought treatment.

A set of 534 spring barley (*H. vulgare* ssp*. vulgare*) MAGIC DH lines were randomly chosen from an eight‐way cross population constructed by intercrossing eight barley genotypes, including one plant from each of seven landraces, Ackermanns Bavaria IPK No. HOR 100 (Ack. Bavaria), Ackermanns Danubia IPK No. BCC 1427 (Ack. Danubia), Criewener 403 IPK No. HOR 62, Heils Franken IPK No. BCC 1433, Heines Hanna IPK No. HOR 59, Pflugs Intensiv IPK No. BCC 1441, and Ragusa IPK No. BCC 1359, and the elite cultivar Barke as described by Sannemann et al. (2015). Ragusa is a six‐rowed facultative barley and other parents are two‐rowed spring barley ecotypes. To study the effect of genetic mechanisms involved in plant development and flowering time on grain yield components under TD, seven above‐ground yield‐related traits were evaluated under WW and TD treatments. MAGIC DH lines and a set of controls consisting of eight parents and spring barley varieties were sown into 19.5 × 25.5 cm plastic pots filled with 5.5 L of Terrasoil®. For each treatment, four seeds per genotype were sown in each pot on April 4 (mean temperature: 11.99°C) and April 3 (mean temperature: 12.51°C) in 2011 and 2012, respectively. An augmented experimental block design was used under foil tunnel which consisted of 20 varieties as checks with replicates every 20 pots at Campus Poppelsdorf (50°43′34.1″N; 7°05′14.6″E) of University of Bonn, Institute of Crop Science and Resource Conservation, Chair of Plant Breeding. Daily temperature from sowing to the end of growth season was recorded for each year. Daily minimum, mean, and maximum temperature for growth season in each year are presented in Data S1. The permanent wilting point was determined at 1.5 MPa matric potential (*θ*_−1.5_) using pressure outflow apparatus procedure as described by Tolk (2003). The pots were irrigated three times a day (6:15 a.m., 12:15 p.m., and 6:15 p.m.) using a computer‐mediated drip irrigation system to keep the volumetric water content (VWC) at 40%. VWC was measured by a HOBO Soil Moisture Smart‐Sensor S‐SMB‐M003 (Onset, MA, USA) in pot, and eight sensors were evenly distributed across whole experiment. The reduction in irrigation started from 35 days after sowing (DAS) when plants were at BBCH 20‐32 (beginning of tillering—positioning of node 2 of main stem at least 2 cm above node 1) in 2011 and BBCH 22‐30 (detecting two tillers—beginning of stem elongation) in 2012 which was 9 and 13 days before start of heading for years 2011 and 2012, respectively, for TD treatment. The treatment was imposed for 5 weeks in three stages. During the first 21 days, the water content in the pots was reduced to the permanent wilting point (15% VWC); then for 8 days, the water content was stabilized at 15% VWC; at 65 DAS, the pots under TD were rewatered slowly to 30% VWC; and finally at 73 DAS, the pots were rewatered to 40% VWC. For WW, VWC was sustained at 40% throughout the experiment (Figure 1).

### 2.2 Phenotypic traits

The phenotypic values for days to heading (DHE), grain filling period (GFP), PLH, above‐ground biomass (AGB), number of ears (NE), number of kernels (NK), and thousand kernel weight (TKW) were measured as described in Table 1. For evaluating ST for traits that were affected by TD, STI was calculated for each DH line in each year using the following equation:

$$\mathrm{STI}=\frac{\;y_{p}\times y_{s}\;}{\;{\left({ȳ}_{p}\right)}^{2}\;},$$

where *y*_*p*_ is the trait for genotype under WW, *y*_*s*_ is the trait for genotype under TD, and *ȳ*_*p*_ is the trait mean for genotype under WW (Fernandez, 1992).

**TABLE 1:** Descriptive statistics and heritability (*H* ^2^) under WW and TD treatments for spring barley MAGIC population.

					Parents		MAGIC population					
Trait	Description	Methods of measurement	Unit	Treat	Mean	Min	Max	Mean	SE	*SD*	CV	*H* ^2^
DHE	Days to heading	Number of days from sowing until emergence of 3 cm of awns	Days	WW	54.90	44.00	76.00	56.87	0.15	5.00	8.79	0.73
				TD	55.10	44.00	76.00	56.80	0.15	4.78	8.40	0.75
GFP	Grain filling period	Number of days from heading to hard dough ripening	Days	WW	38.20	8.00	53.00	38.50	0.10	3.38	8.79	0.23
				TD	38.00	10.00	65.00	37.76	0.11	3.47	9.21	0.27
PLH	Plant height	Distance between soil ground level and tip of awns in cm on 88 DAS	Cm	WW	95.90	44.00	140.00	92.16	0.48	15.75	17.09	0.54
				TD	85.00	52.00	119.00	79.53	0.31	10.27	12.92	0.66
AGB	Above‐ground biomass	Amount of dry above‐ground biomass	g/plant	WW	11.60	1.26	19.15	12.00	0.09	3.00	24.05	0.28
				TD	6.70	2.04	11.24	6.74	0.06	1.88	27.97	0.002
NE	Number of ears	Number of ripe ears	No/plant	WW	4.20	0.50	11.25	5.31	0.05	1.59	29.82	0.33
				TD	2.90	1.00	10.00	3.43	0.04	1.30	37.88	0.24
NK	Number of kernels	Amount of grains per ear	No/ear	WW	25.50	1.65	73.33	21.06	0.20	6.40	30.39	0.70
				TD	20.80	2.25	46.00	16.96	0.14	4.70	27.73	0.59
TKW	Thousand kernel weight	Weight of 1000 grains	Gram	WW	52.20	28.57	64.46	49.65	0.20	6.38	12.86	0.69
				TD	50.30	17.03	69.87	46.81	0.15	4.97	10.62	0.70

Abbreviations: CV, coefficient of variation (standard deviation divided by mean); DAS, days after sowing; MAGIC, multiparent advanced generation intercross; *SD*, standard deviation in percent; SE, standard error; TD, late‐terminal drought; WW, well‐watered.

### 2.3 Statistical analysis

#### 2.3.1 Analysis of phenotypic data

Descriptive statistics were calculated for seven traits across 2 years by using summary function from core generic functions of R software to determine minimum, maximum, and mean. Functions std.error, var, and sd were used to calculate standard error, standard deviation, and coefficient of variation (CV), respectively, using R software (R core team, 2015). Analysis of variance (ANOVA) was performed for each trait using the restricted maximum likelihood (REML) method by PROC MIXED procedure in SAS (9.4 version, SAS Institute Inc., Cary, NC, USA) to fit the following mixed model:

Yijk=μ+Li+Tj+Ck+Li×Tj+εijk,

where *Yijk* is the response variable; *μ* is the general mean; *Li* is the random effect of *i*th DH line; *Tj* is the fixed effect of *j*th treatment; *Ck* is the random effect of the *k*th calendar year; *Li × Tj* is the random effect of interaction of *i*th DH line with *j*th treatment, and *εijk* is the residual.

Variance components were estimated for each treatment by taking genotype (nonreplicated MAGIC DH lines), year, and genotype × year interaction as random effects using REML method by PROC VARCOMP in SAS. Then, broad sense heritability (*H* ^*2*^) for each trait was estimated as

$${H^{2}}_{\left(\text{well}-\text{watered or drought}\right)}=\frac{V_{G}}{V_{G}+\frac{V_{\mathrm{GY}}}{y}+\frac{V_{E}}{y}},$$

where *V*_*G*_ is the variance of genotype; *V*_*GY*_ is the variance of genotype × year; *V*_*E*_ is the variance of experimental error; and *y* is the number of years.

Effect of row and column was evaluated separately for WW and TD treatments in R software with lm function to fit a fixed model by taking nonreplicated MAGIC DH lines, row, column, and controls as fixed effects. LSmeans was calculated for each trait using lsmeans function from “emmeans” package in R and then was used to calculate correlations among traits for each treatment. Correlation coefficients were computed by the Pearson's coefficient (*r*) using rcorr function from package “Hmisc” in R software.

#### 2.3.2 Marker–trait association analysis

For marker–trait association analysis, this study used available set of 5199 single nucleotide polymorphism markers (SNPs) with minor allele frequency (MAF) ≥ 1% and <10% missing values from Afsharyan et al. (2020) which described handling of missing data and MAF. This data set was produced by genotyping 534 spring barley MAGIC DH lines by Illumina 9k iSelect SNP array (Comadran et al., 2012) processed by Sannemann et al. (2015). The analysis of allelic variation was performed to distinguish the contribution of each parent in the observed traits using haplotype data described by Afsharyan et al. (2020) (MAF ≥ 1%) which included 4557 SNP markers with ≤15.5% missing haplotype‐phase values. The population did not show significant structure (Sannemann et al., 2015). Due to unreplicated experiment design, the Best Linear Unbiased Prediction values for each treatment in each year were produced separately using lmer function from package “lme4” in R software (R Core Team, 2015). The analysis was performed using REML to fit a mixed model by considering nonreplicated MAGIC DH lines as random effects. Then, ranef function from the same package was used to estimate Best Linear Unbiased Prediction values for QTL analysis under WW and TD. Values for drought tolerance index was used as phenotypic input to determine the loci associated with ST for each respective trait.

The QTL analysis was performed separately for WW, TD, marker by treatment interaction (M × T), and ST using the PROC MIXED procedure in SAS 9.4 using the following linear model:

Yij=μ+Mi+Tj+Mi×Tj+εij,

where *Yij* is the response variable; *μ* is the general mean; *Mi* is the fixed effect of *i*th marker; *Tj* is the fixed effect of the *j*th treatment (only included for M × T analysis); *Mi × Tj* is the fixed interaction effect of *i*th marker with *j*th treatment (only included for M × T analysis), and *εij* is the residual. The possibility of detecting false positive QTL was addressed by implementing multilocus analysis (Bauer et al., 2009; Kilpikari & Sillanpää, 2003; Sillanpää & Corander, 2002). This approach is a forward selection procedure that in each iterative cycle inserts the most informative SNP inside the model and uses it to reanalyze the remaining SNPs. The iteration continues until no other SNP is found. *P*‐values were calculated by *F*‐tests and probability of false discovery rate was used to adjust *P*‐values by incorporating the control of the QTL false discovery rate (false discovery rate value ≤.05) inside the model using PROC MULTTEST procedure. The model defined the significance of the SNPs for main effects as well as M × T by a threshold of *P*‐value ≤ 0.001 with 1000 permutations and false discovery rate value ≤.05 as putative QTL for the next iteration (Doerge & Churchill, 1996). The significance of QTL was validated by calculating the mean *P*‐value of 20 rounds of a “leave20%out” cross‐validation procedure. The codes and procedure regarding QTL analysis including M × T analysis was described in detail by Afsharyan et al. (2020). The QTL intervals were defined in the model by clustering SNPs based on their significance in the first iteration of the multilocus procedure. The confidence interval was set as 3.5 cM on both sides of the most significant SNP marker based on linkage disequilibrium of the population (Sannemann et al., 2015). Genetic variance explained by a single SNP marker (*R*_*M*_^*2*^) was conducted as

$${R_{M}}^{2}={\mathrm{SQ}}_{M}/{\mathrm{SQ}}_{g},$$

where *SQ*_*M*_ is the sum of squares of *M* and *SQ*_*g*_ is the type I sum of squares of the barley MAGIC DH lines in an ANOVA model (Von Korff et al., 2006). Additionally, total explained genetic variance by all QTL was determined.

Based on population linkage disequilibrium, a window of 7 cM was determined to compare detected QTL positions that colocate in the same region as known genes/QTL described in literature that used the same genetic map and markers (Afsharyan et al., 2020; Alqudah et al., 2014, 2016, 2018; Comadran et al., 2012; Mathew et al., 2018; Maurer et al., 2015, 2016; Pham et al., 2019; Sannemann et al., 2015). In silico analysis was performed using the IPK barley BLAST server (Colmsee et al., 2015). Analysis of allelic variation was performed by using haplotype data as input for marker–trait association analysis as described by Afsharyan et al. (2020).

## 3 RESULTS

### 3.1 Evaluating traits under WW and TD treatments in MAGIC DH lines

**FIGURE 2:**
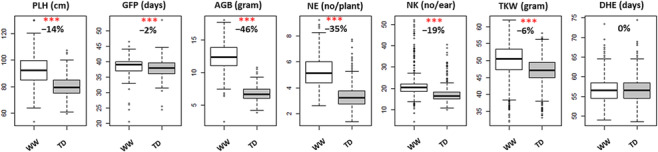
Box–whisker plots describe the variation for six traits affected by treatment in 534 spring barley multiparent advanced generation intercross population under WW and TD treatments using mean values for 2011 and 2012. Trait names and units are indicated above their respective subplot. Significant treatment effect is indicated with red asterisks with *P < 0.05, **P < 0.01, and ***P < 0.001. Change in trait mean (%) under TD treatment compared with WW is shown below the asterisks. AGB, above‐ground biomass; DHE, days to heading; GFP, grain filling period; NE, number of ears; NK, number of kernels; PLH, plant height; TD, late‐terminal drought; TKW, thousand kernel weight; WW, well‐watered.

To study seven yield‐related traits under WW and TD, a set of 534 spring barley MAGIC DH lines were used in a pot experiment under foil tunnel in two consecutive years. The start of reducing irrigation was shortly before flowering at 35 DAS (Figure 1). ANOVA revealed that TD effect was significant (*P* < 0.001) for all traits except for DHE (Table S1) and, depending on the trait, showed a reduction ranging from 2% to 46% under TD (Figure 2). DH lines exhibited lower CV under TD for PLH, NK, TKW, and DHE; while for GFP, NE, and AGB, the variation was higher under TD. Heritability (*H* ^2^) was estimated to be >50% for DHE, PLH, NK, and TKW in both WW and TD (Table 1). Correlation coefficients (Table S2) revealed higher correlation between AGB and PLH (*r* = 0.69) as well as AGB and NE (*r* = 0.66) under WW and between AGB and NE (*r* = 0.63) under TD. The highest CV for drought ST belonged to NK‐ST and the lowest was for GFP‐ST. The heritability ranged from 21% for AGB‐ST to 79% for NK‐ST (Table S3). Correlations coefficients for ST were higher for NE‐ST and AGB‐ST (*r* = 0.65) (Table S4).

### 3.2 Marker–trait associations under WW and TD treatments

**FIGURE 3:**
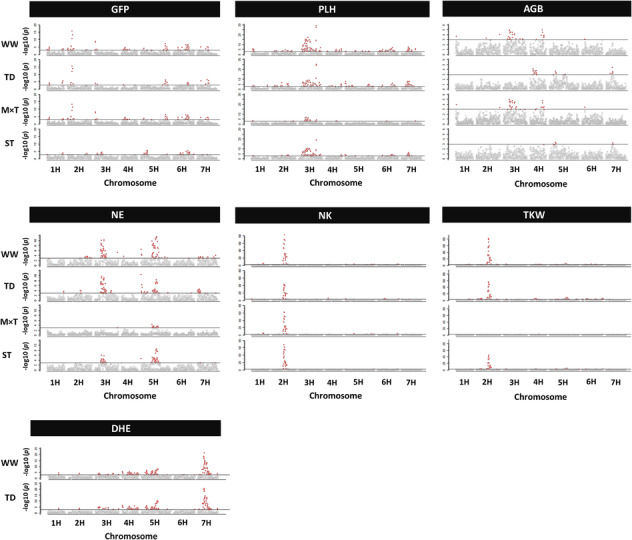
Manhattan plots describe quantitative trait loci analysis of seven traits for WW treatment, TD treatment, M × T, and drought tolerance using the spring barley multiparent advanced generation intercross population. The *y*‐axes denote the significance of SNP markers as −log10 (*P*); the chromosomes are denoted on the *x*‐axes. The highlighted SNP markers above the cutoff line are significant by a threshold of *P* ≤ 0.001 with 1000 permutations plus 20 times cross‐validation. AGB, above‐ground biomass; DHE, days to heading; GFP, grain filling period; M × T, marker × treatment interaction; NE, number of ears; NK, number of kernels; PLH, plant height; ST, stress tolerance; TD, late‐terminal drought; TKW, thousand kernel weight; WW, well‐watered.

**FIGURE 4:**
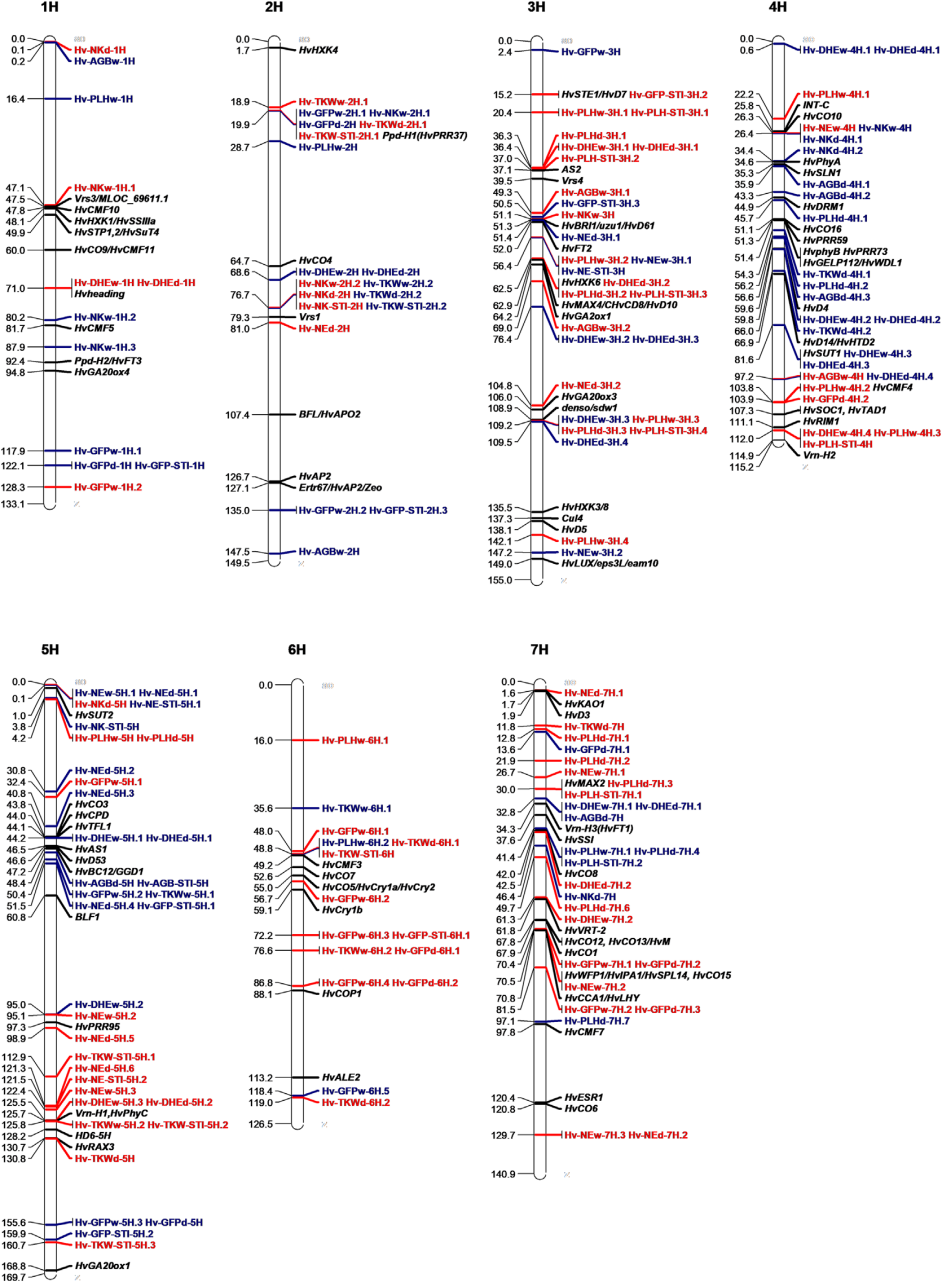
Genetic map of quantitative trait loci (QTL) detected for traits days to heading, grain filling period, plant height, above‐ground biomass, number of ears, number of kernels, and thousand kernel weight under well‐watered and late‐terminal drought treatments as well as stress tolerance in spring barley multiparent advanced generation intercross DH lines. Barley chromosomes are exhibited with white bars. The position for the peak SNP marker represents each QTL which is colored according to increasing (red) or decreasing (blue) effect of the minor allele of QTL. The located QTL are detailed in Tables S5, S6, and S8. The names of known genes as described for the Barke × Morex recombinant inbred lines (RILs) by Mascher et al. (2013) are italicized in black indicating their position on chromosomes.

Genetic analysis was performed to identify the associated genetic regions under WW and TD for each trait (Figure 3). Results showed that some of genetic regions harbored QTL for various traits or treatments such as the region on chromosome 2H (18.90–28.70 cM), but others contained trait and/or treatment specific QTL such as locus on chromosome 1H (71.03 cM) for flowering time and locus on chromosome 4H (34.4–35.9 cM) for TD which was associated with NK and AGB (Figure 4). In total for the seven traits, 69 and 64 QTL were found for WW (Table S5) and TD (Table S6), respectively. The number of total loci associated with each trait under both treatments ranged from 10 QTL for AGB to 25 QTL for PLH. Genetic variation explained by detected QTL for each trait under WW ranged from 11.29% for AGB to 60.10% for NK and under TD spanned from 8.71% for AGB to 52.88% for TKW. Among investigated traits, DHE was mostly controlled by the same genetic regions under WW and TD as it was not affected by treatment. For majority of the traits, the most significant QTL were located at the same loci under both treatments. The prominent regions harboring QTL was located on chromosome 2H for GFP (BK_12 at 19.90 cM), NK and TSW (SCRI_RS_165473 at 76.66 cM), 3H for PLH (SCRI_RS_103215 at 109.21 cM), and 7H for DHE (BOPA1_12701_485 at 32.79 cM). The most significant QTL for AGB was detected on 4H chromosome (BOPA2_12_30718 at 97.17 cM) under WW and on 7H (BOPA1_12701_485 at 32.79 cM) under TD and, for NE, on 5H chromosome at 122.43 cM for WW (BOPA2_12_21471) and at 0.14 cM for TD (BOPA1_3417_1451). The results revealed QTL associated with GFP, NK, AGB, TKW, NE, and PLH which were detected only under TD. Analysis of M × T identified the loci that have significant difference in allelic effects among treatments (Figure 3). The results revealed overall 25 genetic regions interacting with treatment for four traits which ranged from 10 for GFP to four for PLH (Table S7). The results included prominent QTL for GFP (BK_12 at 2H, 19.90 cM) and NK (SCRI_RS_165473 at 2H, 76.66 cM) which colocated with flowering time locus *Ppd‐H1* and grain number locus *Vrs1*, respectively. The most significant locus involved in treatment interaction for AGB (BOPA1_2838_663 at 3H, 49.29 cM) and PLH (SCRI_RS_173916 at 3H, 51.20 cM) colocated with PLH locus uzu1 (Table S7).

Chromosomal regions associated with drought tolerance were evaluated for each trait that was affected by treatment (Figure 3). In total, 25 QTL were identified (Table S8) from which 21 colocalized with QTL for the respective traits under either or both treatments. For GFP‐ST, seven QTL were detected that explained a total genetic variation of 28.59%. Three QTL on chromosome 1H (122.10 cM), 5H (159.93 cM), and 6H (72.17 cM) colocalized with loci detected for WW, TD, and M × T. All estimated seven QTL regions for PLH‐ST explained 36.57% of genetic variation. Among them, loci on 3H (109.21 cM), 7H (41.43 cM), and 3H (62.54 cM) colocalized with QTL detected for all or either of WW, TD, and M × T. Three QTL were found for NE‐ST that explained 17.51% of genetic variance, and all were commonly located for NE under WW and TD. QTL analysis for NK‐ST revealed two regions including a QTL on 2H (76.66 cM) which coincided with the loci found for WW, TD, and M × T and another on 5H (3.82 cM) that colocated with region found for TD. For TKW‐ST, six positions were located from which four on 2H (19.90 cM), 2H (76.66 cM), 5H (125.76 cM), and 6H (48.80 cM) colocalized with either or both WW and TD. No QTL regions was found for AGB‐ST which was expected, as genotypic variation for AGB‐ST was not significant (*P* < 0.001).

**FIGURE 5:**
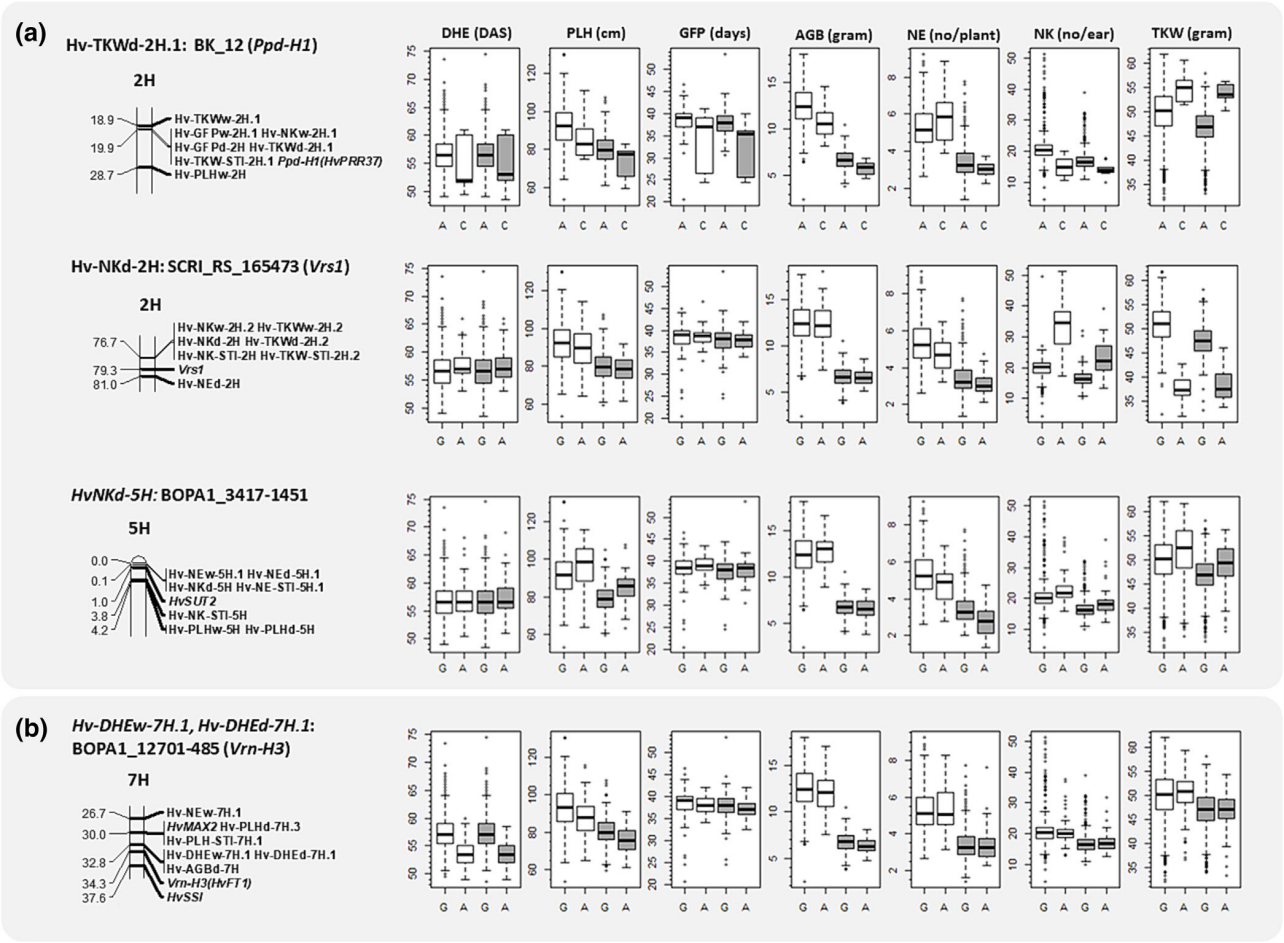
Distribution of trait values under both treatments for prominent quantitative trait loci (QTL) showing favorable effect on grain number or grain weight under late‐terminal drought (a) as well as the most prominent flowering time QTL (b). On the left side, the name of the QTL, prominent SNP marker, and chromosomal position including all other QTL commonly located in the region are shown. Italicized gene names indicate the major gene that the QTL colocated with according to their position as described for the Barke × Morex RILs by Mascher et al. (2013). Box–whisker plots illustrate distribution of trait values for the major and minor alleles of the most prominent SNP marker of the QTL for all yield‐related traits under well‐watered (white) and late‐terminal drought (gray) treatments. The trait values are represented in *y*‐axis, and the major and minor alleles are represented in *x*‐axis. The traits and trait units are indicated above the first row of subplots. Described minor alleles are originated from single parent, and their favorable effect is estimated to be >.5 trait unit compared with population mean. The located QTL are described in Tables S5 and S6. AGB, above‐ground biomass; DHE, days to heading; GFP, grain filling period; NE, number of ears; NK, number of kernels; PLH, plant height; TKW, thousand kernel weight.

QTL analysis of grain yield components, NE, NK, and TKW under TD revealed loci with desirable effect (Table S6), including three QTL that showed allelic effect of >0.5 trait unit and were derived from parental line Ragusa (Figure 5a and Table S6). Among them was QTL allele that revealed desirable effect for TKW (2H, 19.90 cM; BK_12) colocated with flowering time locus *Ppd‐H1* and the underlying allele from Ragusa showed an increasing effect of 6.23 g compared with population average. This locus was also found for TKW‐ST. Analysis of allelic variation showed that the alleles from Bavaria, Ack. Danubia, Criewener/Pflugs Intensiv, and Heines Hanna had negative effect, and among them, Criewener/Pflugs Intensiv had the most decreasing effect by −0.8 g compared with the population average. A novel QTL allele for NK (BOPA1_3417_1451 at 5H, 0.14 cM) explained 3.91% of genetic variance (Table S6). The allele underlying this QTL showed 0.65 increase for grain number per ear compared with the population mean. This locus was also detected for NE under both treatments. A QTL was found near this locus for NK‐ST. The allele originating from parental lines Ack. Danubia, Barke, Criewener/Pflugs Intensiv, and Heines Hanna showed decreasing effect, and the most negative effect was for Criewener/Pflugs Intensiv by −0.7 grain number per ear compared with the population average. One other QTL allele for NK (SCRI_RS_165473 at 2H, 76.66 cM) showed that allele from Ragusa improved grain number per ear by 2.50 compared with the population mean. It was located near grain number locus *Vrs1*. Analysis of allelic variation showed that the alleles from other parental lines had decreasing effect with Barke having the most negative effect by −0.5 grain number per ear compared with the population average. This locus was also detected for M × T and NK‐ST. M × T analysis identified this region and validated the different effects detected for this locus under WW and TD which was in line with results from NK‐ST that revealed its association with drought tolerance.

The loci for all identified QTL (WW, TD, and ST) were compared in a window of 7 cM to determine genetic regions that controlled various traits (Figure 4 and Tables S5, S6, and S8). The findings revealed 15 common regions linked to at least three traits. The locus that was populated the most was located on 5H (44.24–51.46 cM) and was associated with five traits including DHE (WW and TD), GFP (WW, TD, and ST), AGB (TD and ST), NE (TD), and TKW (WW and ST).

## 4 DISCUSSION

### 4.1 Effect of TD on spring barley

Spring barley MAGIC DH lines were investigated for seven traits under WW and TD in pot experiment under foil tunnel. The drought treatment started before start of heading and spanned from 35 to 73 DAS which overlapped with the duration of heading for the population (Figure 2). However, all traits showed decrease under TD except for DHE, possibly due to imposing TD shortly before start of heading. During the intense stage of drought treatment from 56 to 65 DAS, majority of genotypes reached to grain filling and seed development stage (Figure 2), exposing them to TD (Samarah et al., 2009; Samarah & Alqudah, 2011). Major reasons for reduction in NE and grains of barley during drought are increased sterile ears per plant (Mogensen, 1992; Samarah, 2005; Sánchez‐Díaz et al., 2002) and loss in seed set (Del Moral et al., 2003; González et al., 1999; Mogensen, 1992). As a result of timing of treatment, number of sterile fully developed florets did not decrease; therefore, loss in grain number could be due to grain abortion (González et al., 2003; Miralles et al., 2000). Height reduction in MAGIC DH lines might be the result of decreased gross photosynthetic rate and osmotic potential (González et al., 1999; Hopkins & Wilhelmova, 1997; Taiz & Zeiger, 2002). TD most impacted AGB which was positively correlated with PLH (González et al., 1999; Sánchez‐Díaz et al., 2002) and NE. Severe impact of drought treatment on above‐ground biomass was reported in previous studies (Pham et al., 2019; Samarah et al., 2009). Reduction of NK, NE, TKW, and GFP in MAGIC DH lines was strong indication of yield loss (Samarah et al., 2009). On the other hand, positive correlation of TKW with GFP was smaller and in opposite direction with NK and NE, suggesting rise in source‐sink ratios (Serrago et al., 2013) led to increase in weight of single grain which partly compensated the negative effect of shorter GFP. Due to the possible effect of pot size on root growth, further studies in field condition are required.

### 4.2 QTL for WW and TD treatments and drought tolerance

Genetic regions associated with the traits for WW, TD, M × T, and ST were detected on all chromosomes of barley (Figure 3). The total 5199 SNPs (<0.01 MAF) provided an approximate coverage of 1 SNP per 0.191 cM which detected QTL coinciding with known loci as well as new regions for all the traits used for four marker–trait association approaches. The barley 9K Illumina SNP array has been successfully used in various QTL mapping studies in different types of barley population (Alqudah et al., 2014, 2016; Maurer et al., 2015, 2016; Pham et al., 2019; Sannemann et al., 2015). A recent study for QTL mapping under drought condition using the same SNP array reported successful QTL mapping for several traits with the marker density of 1 SNP per 0.186 cM (Pham et al., 2019) which shows that the SNP coverage provided by the SNP array in the current study is sufficient for the analysis. The majority of QTL coincided with known gene/QTL regions with high precision (Tables S5–S8). Peak marker BK_12 on chromosome 2H is gene‐specific for *Ppd‐H1* gene (Colmsee et al., 2015) and was detected for traits GFP (WW, TD, and M × T) and TKW (TD and ST) which were reported to be associated with *Ppd‐H1* region (Honsdorf et al., 2017; Maurer et al., 2016). A previous study also reported detecting the same marker for flowering time, which is known to be regulated by *Ppd‐H1* gene, as an indication of QTL mapping precision in another multiparental population (Maurer et al., 2015). The causative allele for loci corresponding to *Ppd‐H1* gene is inherited from Ragusa, and this locus was detected for WW (SCRI_RS_233272, 2H 18.91 cM), TD (BK_12, 2H 19.90 cM), and ST (BK_12, 2H 19.90 cM). The results for estimating allelic effect validated the desirable effect of allele from Ragusa and also showed that allele from parent Criewener/Pflugs Intensiv had the least desirable effect (Tables S5, S6, and S8). Another well‐known locus *Vrs1* (*Six‐rowed spike 1*) which controls grain number in inflorescence (Komatsuda et al., 2007) aligned with QTL for yield component traits TKW (WW, TD, and ST), NK (WW, TD, M × T, and ST), and NE (TD), and the causative allele is also inherited from Ragusa which is the only six‐rowed parental line (Figure 4). *Vrs1* allele inherited from six‐rowed barley is expected to have a stronger increasing effect for NK compared with other two‐rowed parental lines as well as the opposite effect on TKW as higher grain number is expected to induce competition among single grains for assimilates (Maurer et al., 2016).

Even though this locus is explaining a large amount of phenotypic variance for NK and TKW under WW and TD, the QTL analysis was sufficiently powerful to reveal other loci including drought specific QTL with favorable affect such as loci on chromosomes 4H (34.35) and 5H (0.4 cM) for NK as well as on chromosome 4H (54.32 and 66.01 cM), 6H (48.80 and 118.98 cM), and 7H (11.83 cM) for TKW. The allelic variations of eight parental lines used in the MAGIC strategy were evaluated to provide an overview of contribution for each parent; for example, locus detected on 6H (118.98 cM) for TKW under TD had alleles originated from Ragusa (1.804 g), Heils Franken (1.5 g), and Barke (0.002) that showed increasing effect while Bavaria (−0.608 g), Criewener/Pflugs Intensiv (−0.355 g), Danubia (−0.968 g), and Heines Hanna (−1.216 g) showed reducing effect (Table S6).

QTL analysis results for ST and M × T coincided in a number of regions, and the majority of the QTL that did not overlap could be detected under WW and TD. Majority of QTL found for ST colocated with at least one QTL for WW, TD, and M × T. Reported QTL mapping attempts for drought tolerance produced small marker–trait association effects (Abdel‐Haleem et al., 2012; Li et al., 2013; Sallam et al., 2019; Wehner et al., 2015) that could be missed (Honsdorf et al., 2014). However, this study successfully detected regions including ones harboring known genes. Analysis of QTL for ST revealed QTL associated with drought tolerance and analysis of M × T identified QTL that have significant difference in allelic effects between treatments (Courtois et al., 2000) which collectively revealed a higher number of regions and can give more insights on complex mechanism of polygenic yield‐related traits under drought in barley. Results of M × T could be informative on whether a QTL can be selected for breeding purpose regardless of treatment. Therefore, ST loci commonly detected for WW and TD that were not identified by M × T such as QTL on 2H chromosome 19.90 cM (*Ppd‐H1*) for TKW could be investigated to improve the traits independent of the treatment (Figure 4).

Association of a number of loci with multiple traits suggested their pleiotropic effect. For instance, *Ppd‐H1* region which is reported to be linked with GFP under normal condition (Maurer et al., 2016) and TD (Honsdorf et al., 2017) was detected for GFP (WW, TD, and ST), TKW (WW, TD, and ST), and PLH (WW) in this study (Figure 4). The region reported to be linked with dry weight under control and drought stress condition (Pham et al., 2019) which harbors semi‐dwarfing gene semi‐brachytic 1 (*uzu1*) (Saisho et al., 2004) and flowering time gene *HvFT2* (Faure et al., 2007) colocated with QTL for GFP (ST), PLH (M × T), AGB (WW and M × T), and NE (WW, TD, and ST) and revealed treatment interaction for PLH and AGB (Tables S5, S6, and S8).

Flowering time loci reported for this population including *Vrn‐H1* and *Vrn‐H3* (Afsharyan et al., 2020; Mathew et al., 2018; Sannemann et al., 2015) were found for DHE (WW and TD). More QTL were detected for DHE compared with Sannemann et al. (2015) which utilized different methods for data preparation and QTL analysis. QTL for PLH (WW, TD, M × T, and ST) corresponded to semidwarf locus (*denso/sdw1*) which is reported to be associated with height under drought (Pham et al., 2019) and normal condition (Maurer et al., 2016; Pham et al., 2019). Overall, a number of 26, 20, and 11 loci were identified for WW, TD, and ST, respectively, for various traits that could not be linked to a known QTL.

### 4.3 QTL with favorable effects on grain weight, grain number, and ear number under TD

One QTL for TKW and two for NK, inherited from a single parent, showed desirable effect >0.5 trait unit and were commonly detected for drought tolerance (Figure 5a and Tables S5, S6, and S8). These QTL were positioned in loci harboring genes involved in developmental mechanisms. The QTL for TKW colocated with major flowering time locus *Ppd‐H1*. However, this region was not detected for flowering time in this study (Afsharyan et al., 2020; Sannemann et al., 2015), and effect of the most prominent QTL for flowering time which colocated with another major gene *Vrn‐H3* did not correspond to effect detected for *Ppd‐H1* locus (Figure 5b). This region was reported to be mapped for thousand grain weight under drought treatment that was imposed after flowering time (Honsdorf et al., 2017). Therefore, the results suggest *Ppd‐H1* might improve drought tolerance and grain weight by ways other than timing of flowering. The allele underlying this locus was inherited from parental line Ragusa and showed 6.23 g increase in grain weight (Figure 4). The QTL allele for NK, located on 5H chromosome (0.14 cM), was not reported before. This region was also mapped for NE under WW, TD, and for ST which was in line with reports that found it linked with tiller number in control (Alqudah et al., 2016) and drought stress condition (Pham et al., 2019). The far distal region of 5H chromosome was reported to be associated with grain size parameters (area, width, and diameter) and grain filling rate in normal field condition (Du et al., 2019). NK and NE were negatively correlated which corresponded to opposite effect of this locus on grain number and ear number (Figure 5a and Table S6). The allele underling this QTL is inherited from parental line Ragusa and shows a desirable allelic effect of 0.65 increase for number of grains per ear for NK. This NK QTL allele is nearby sucrose transporter gene *HvSUT2* which is involved in carbohydrate metabolism and encodes sucrose transport protein in different organs including endosperm of developing grains in barley (Weschke et al., 2000). It was reported that restricted carbohydrate supply might lead to grain abortion in water‐stressed maize (Zinselmeier et al., 1999). Therefore, the results suggests that sugar‐related genes might contribute to controlling grain number which in barley is reported to be majorly controlled by *Vrs1* gene (Komatsuda et al., 2007). *Vrs1* locus is known to control spike row number as well as affecting traits related to grain size and weight (Ayoub et al., 2002; Sharma et al., 2018; Wang et al., 2016, 2017). This locus showed that the causative allele from Ragusa had favorable effect to increase grain number per ear by 2.50. However, the increase in grain number might have a negative influence on grain weight (Calderini et al., 2021; Maurer et al., 2016), and the impact of the trade‐off should be considered on the potential yield. The findings indicated that spring barley MAGIC population can provide insights to reveal favorable QTL alleles to improve drought tolerance and grain yield under drought stress in breeding programs.

## 5 CONCLUSION

Grain yield in barley under drought is a complex trait due to contribution of different developmental and yield‐related mechanisms. Using spring barley MAGIC population in this study successfully identified known and newly reported loci associated with regulation of grain yield and drought tolerance under TD. Multiple QTL with favorable effects on grain yield components were detected on chromosomal regions harboring genes for flowering time and developmental traits including one located on the major flowering time gene *Ppd‐H1* for thousand grain weight as well as novel QTL allele on 5H chromosome for grain number per ear. These findings indicate that contribution of flowering time and developmental mechanisms to grain yield can be examined in future to explore strategies for yield improvement in barley under drought stress.

## AUTHOR CONTRIBUTIONS

NPA was involved in conceptualization, methodology, statistics and QTL mapping, visualization, interpretation and discussion, writing—original draft, and writing—review and editing. WS was involved in conceptualization, methodology, foil tunnel experiment and phenotyping, and writing—review and editing. AB was involved in conceptualization, methodology, and writing—review and editing. JL was involved in conceptualization, methodology, supervision, project administration, funding acquisition, and writing—review and editing. All the authors have read and approved the final manuscript.

## CONFLICT OF INTEREST STATEMENT

The Authors did not report any conflict of interest.

## PEER REVIEW

The peer review history for this article is available in the Supporting Information for this article.

## Supporting information


**Supporting Information S1.** Supporting Information

**Data S1** Daily temperature (°C) for growth season in 2011 and 2012 (Minimum, Mean, Maximum)

**Tables S1** Analysis of variance for 534 MAGIC DH lines showing F and *P* values
Tables S2 Pearson correlation coefficient (r) for traits using LSmeans under well‐watered (dark grey, italinazed) and late‐terminal drought (light grey) treatments in spring barley MAGIC population.
Tables S3 Descriptive statistics, heritability (*H2*) for drought stress tolerance (ST) for six traits in spring barley MAGIC population
Tables S4 Pearson correlation coefficient (r) for drought stress tolerance index (ST) of six traits measured in spring barley MAGIC population.
Tables S5 Significant QTL for seven traits under well‐watered treatment in spring barley MAGIC population
Tables S6 Significant QTL for seven traits under late‐terminal drought treatment in spring barley MAGIC population
Tables S7 QTL × Treatment interactions (P‐value <0.1E‐3) via cross‐validated multi‐locus QTL analysis of six traits in spring barley MAGIC population
Tables S8 Significant QTL for drought stress tolerance (ST) of six traits in spring barley MAGIC population

## Data Availability

Data described in this manuscript are available upon request to the author.
