# Supplementary material for: Identifying developmental QTL alleles with favorable effect on grain yield components under late‐terminal drought in spring barley MAGIC population
Source: Plant Direct. 2023 Aug 2;7(8):e516. doi: 10.1002/pld3.516 (PMC10394678; doi:10.1002/pld3.516)
Supplement: Supplementary file 1 — Supporting Information S1. Supporting Information [file PLD3-7-e516-s003.docx]

**DECISION LETTER – Round 1**

May 5, 2023

Dr. Nazanin P Afsharyan
University of Giessen
Heinrich-Buff-Ring 26
Giessen 35392
Germany


RE: Effect of developmental genetic mechanisms on grain yield under late-terminal drought reveals favorable QTL alleles in spring barley MAGIC population

Dear Dr. Afsharyan:

Thank you for submitting to Plant Direct. All required reviews have been returned and we have now finished our evaluation of your manuscript. In light of the reviewers' and editor's comments, further revisions are needed before the paper can be accepted for publication in Plant Direct.

Please view the editors' and reviewers' comments below and use their suggestions as a guide while you work on your revision.

When uploading the revised version of this article, please be sure to include the following:

-A word document that contains your response to the reviewers. You should respond to each reviewer comment and note the changes made to the manuscript. If you do not agree with a reviewer's comment and choose not to make a suggested revision, please explain why. Please try to provide as complete an answer as possible to each reviewer's criticisms.
-A tracked changes document with each change highlighted
- A clean version of the latest version of the manuscript


To upload your revision, please click the link below.
https://plantdirect.msubmit.net/cgi-bin/main.plex?el=A6Lr6Xi3A6noy4I1A9ftdzPS6MnDO7rbYZnHglx3LgZ

In order to provide as timely a service as possible, we ask that your revision is resubmitted within three months after receipt of this request. If an extension is needed, please send a request, along with a brief explanation, to the editorial office at plantdirect@wiley.com .

Please note that, in addition to publishing reviewer comments, the author's responses to review comments will also be published alongside the final version of the paper. If you would not like the author's responses to be published, please contact the editorial office at plantdirect@wiley.com .


Thank you very much for giving us an opportunity to review your work. I look forward to receiving the next version.


Sincerely,

Esther van der Knaap

Esther van der Knaap

Editor, Plant Direct

---------------------------------------------------------------------------- Editor comments:

---------------------------------------------------------------------------- Reviewer comments:
Reviewer #1:

The MAGIC lines were examined by the authors across two seasons (2011 and 2012) in Germany. They have studied grain components and agronomy traits to identify QTLs. Using MAGIC population is appreciable, and known and new loci that have beneficial alleles for TGW and GPE were discovered by the authors. The present study will serve as a valuable source for improving the yield of barley. However, a few more information may be included before publication.
Line 416: a dot after (Figure 3).
Line 439: Ragusa is a 6-row barley so yes I think it is normal that it will induce a huge effect compared to other parents for NK
Line 500: this study did not measure grain yield, only yield components, and a favourable effect to yield component may not warrant an increase in grain yield. So please be careful of using this word as it can mislead readers.
Figure 5: What are G and A, or A and C in the x axis?
Questions: What is the difference in maturity of these lines, or variation of flowering time among lines? Imposing the drought stress onto the plants at different maturity can affect the yields and yield components (ie plants that flower later may be not affected by the drought stress compared to the ones that flowered right when the drought stressed was introduced). Given the large number of line it is hard to introduce drought stress by its growth stage, but the more homogenous maturity will help reduce noise. Please use the data of DHE to elaborate on this.
Suggestions: because the drought stress was introduced primarily before and during the flowering time, is it more precise if we use the term effect of drought stress at reproductive stage? Late-terminal drought seems to give the readers an impression that the drought stress was imposed at grain filling period near harvest.


Reviewer #2:

Afsharyan et al. title "Effect of developmental genetic mechanisms on grain yield under late-terminal drought reveals favorable QTL alleles in spring barley MAGIC population" conducted QTL mapping to uncover genetic mechanisms grain yield formation under late terminal drought stress in barley. This study conducted phenotyping of yield traits in two years 2011 and 2012 using 534 spring barley MAGIC population. The overall objective of this study is to identify genetic loci associated with grain yield-related traits in response to drought stress. This genetic information is crucial to develop drought tolerant barley cultivars. I found this article is very well-studied, methodology and statistical is sound, results are properly presented. There are few minor corrections need to address before considering its for publication. Some specific comments are below:
1. In title "developmental genetic mechanisms" is misleading here. Developmental mechanisms something in-depth pathway-based analysis, while authors only measure some yield attributes. So, title need to modify based on investigated traits and analyses.
2. In Figure 1, author only showed soil moisture content. Moreover, it is also crucial to show temperature fluctuation in the same figure for better understanding. During drought stress, temperature also an important factor.
3. Line 183: please remove treatment from the "well-watered treatment".
4. Line 208: please provide details of the sensor.
5. Why stress tolerance index (STI) is important to study drought responses, provide few sentences of this importance in introduction.
6. Line 259: only ST is enough as it is abbreviated above.
7. In Figure 2, data for DHE is missing.
8. Line 209: Please elaborate BBCH.
9. In discussion, author need to highlight their own findings then justify with other study.
10. This study focused only QTL identification. I found some promising QTL associated with terminal drought is very informative. It would be highly recommended if author can further identify causal genes thus regulated flowering time and yield components.

**DECISION LETTER – Round 2**

June 28, 2023
Nazanin P Afsharyan
University of Giessen
Heinrich-Buff-Ring 26
Giessen 35392
Germany

MSID: 2022-01044R1
MS TITLE: Identifying developmental QTL alleles with favorable effect on grain yield components under late-terminal drought in spring barley MAGIC population

Dear Dr. Nazanin Afsharyan:

I am pleased to inform you that your manuscript "Identifying developmental QTL alleles with favorable effect on grain yield components under late-terminal drought in spring barley MAGIC population" has been accepted for publication in Plant Direct.

Your article will appear online in the next available issue of Plant Direct. To ensure your article gets published as quickly as possible, please pay attention to the steps detailed below. We have found that most of the delays happen at this stage, especially at the payment stage, so please respond as quickly as possible when prompted.

License Agreement: Once your article has been accepted it will move to Production and undergo admin and file checking - you may receive an email with any queries we have at this stage. When all required items are received by the publisher and queries resolved, the corresponding author will receive an email from Wiley's Author Services system which will ask them to log on at https://authorservices.wiley.com/bauthor and will present them with the appropriate license for completion. Your article cannot be published until both the signed license agreement and payment of the article fee have been received.

Payment of the Open Access Article Publication Fee: All articles published in Plant Direct are fully open access: immediately and freely available to read, download and share. Plant Direct charges a publication fee to cover the publication costs. The corresponding author for this manuscript should have already received a quote from the payments team (cs-openaccess@wiley.com) with the estimated article publication fee; please email cs-openaccess@wiley.com if this has not been received. The corresponding author should log on to the Wiley Author Services site, where the publication fee can be paid by credit card or an invoice. Pro Forma can also be requested. Payment of the publication charge must be received before the article will be published online.

Proofs: You will have the opportunity to look over your paper once more when you receive the author proofs for your article. The proofs will be with you in approximately two weeks. Please note that, in addition to publishing reviewer comments, the author's responses to review comments will also be published alongside the final version of the paper. If you would not like the author's responses to be published, please contact the editorial office at plantdirect@wiley.com .

Promotion of your article: You can help your research get the attention it deserves! Check out Wiley's free Promotion Guide for best-practice recommendations for promoting your work at www.wileyauthors.com/eeo/guide. And learn more about Wiley Editing Services which offers professional video, design, and writing services to create shareable video abstracts, infographics, conference posters, lay summaries, and research news stories for your research at www.wileyauthors.com/eeo/promotion.

Thank you again for your contribution to Plant Direct. If you have any questions, feel free to contact the editorial office at plantdirect@wiley.com .

Sincerely,

Esther van der Knaap

Esther van der Knaap

Editor, Plant Direct

---------------------------------------------------------------------------- Editor comments


---------------------------------------------------------------------------- Reviewer comments:

**AUTHORS’ RESPONSE**

Paper number: 2022-01044

Title: Effect of developmental genetic mechanisms on grain yield under late-terminal drought reveals favorable QTL alleles in spring barley MAGIC population

Authors: Nazanin P. Afsharyan, Wiebke Sannemann, Agim Ballvora, Jens Léon

---------------------------------------------------------------------------- Editor comments:

Thank you very much. We revised the manuscript carefully based on the reviewer´s comments and clarified the points mentioned. The page and line numbers mentioned in this response letter are according to the new version of the manuscript.

---------------------------------------------------------------------------- Reviewer comments:

Reviewer #1:

The MAGIC lines were examined by the authors across two seasons (2011 and 2012) in Germany. They have studied grain components and agronomy traits to identify QTLs. Using MAGIC population is appreciable, and known and new loci that have beneficial alleles for TGW and GPE were discovered by the authors. The present study will serve as a valuable source for improving the yield of barley. However, a few more information may be included before publication.

- Thank you very much for your valuable comments and for your positive assessment of our study. We have carefully reviewed your comments and made necessary revisions to improve the clarity of the manuscript. The page and line numbers are according to new version of the manuscript.

Line 416: a dot after (Figure 3).

- Page 14, line 424: It is corrected.

Line 439: Ragusa is a 6-row barley so yes I think it is normal that it will induce a huge effect compared to other parents for NK

-Exactly. The following sentence was added to better highlight this point:

The new version reads:

Page 15, line 443-444: *“…Vrs1 allele inherited from six-rowed barley is expected to have a stronger increasing effect for NK compared to other 2-rowed parental lines …”*

Line 500: this study did not measure grain yield, only yield components, and a favourable effect to yield component may not warrant an increase in grain yield. So please be careful of using this word as it can mislead readers.

- According to the suggestion, “grain yield” was changed to “grain yield components”.

The new version reads:

Page 16, lines 515: “*… QTL with favorable effects on grain yield components …”*

Figure 5: What are G and A, or A and C in the x axis?

- The description was added to figure legend for Figure 5:

The new version reads:

Figure 5: “… *The trait values are represented in y-axis and the major and minor alleles are represented in x-axis.…”*

Questions:

What is the difference in maturity of these lines, or variation of flowering time among lines? Imposing the drought stress onto the plants at different maturity can affect the yields and yield components (ie plants that flower later may be not affected by the drought stress compared to the ones that flowered right when the drought stressed was introduced). Given the large number of line it is hard to introduce drought stress by its growth stage, but the more homogenous maturity will help reduce noise. Please use the data of DHE to elaborate on this.

- Thank you for your comment and for bringing up an important point regarding the overlap between heading and drought treatment. The following boxplot shows the data of days to heading (DHE; trait-unit: days to heading) among lines for well-watered (WW) and late-terminal drought (TD) treatments using mean values for 2011 and 2012:

The drought treatment was imposed on 35 days after sowing which was 9 and 13 days before start of heading for years 2011 and 2012 respectively. At 56 days after sowing (DAS), the soil moisture content was stabilized at 15% VWC and maintained till 65 DAS and then the pots were re-watered to reach soil moisture content 40% VWC at 73 DAS. While, as you noted, it is challenging to impose drought stress at a specific growth stage for a large number of genotypes, we have tried to minimize the potential impact by ensuring that all three stages of drought treatment collectively overlap the duration of heading for the population and as shown by the boxplot for DHE, majority of the genotypes flowered by the end of the second stage which was the intense drought (15% VWC) at 65 DAS. In addition, the effect of treatment for NK, TKW, NE and GFP is significant and all genotypes showed reduced performance for these traits as shown in Figure 2. However, we acknowledge that the drought treatment can not be imposed at an identical developmental stage across all genotypes since all genotypes in a segregating population do not have identical development rate. Therefore, based on your comments, we included a boxplot for DHE in Figure 2 and also added sentences in the discussion to clarify this point. We hope that this will provide a clearer understanding of the variation in flowering time on our study and its overlap with drought treatment.

The new version reads:

The data for DHE was added to Figure 2:

**Figure 2** Box-Whisker plots describe the variation for six traits affected by treatment in 534 spring barley MAGIC population under well-watered (WW) and late-terminal drought (TD) treatments using mean values for 2011 and 2012. Trait names and units are indicated above their respective sub-plot. Trait abbreviations are PLH: plant height, AGB: above ground biomass, GFP: grain filling period, NE: number of ears, NK: number of kernels, TKW: thousand kernel weight, DHE: days to heading. Significant treatment effect is indicated with red asterisks with **P* <0.05, ***P* <0.01 and ****P* <0.001. Change in trait mean (%) under terminal drought treatment compared to well-watered is shown below the asterisks.

The new version reads:

Page 14, lines 404-405: *“…The drought treatment started before start of heading and spanned from 35 DAS to 73 DAS which overlapped the duration of heading for the population (Figure 2).…*”

Suggestions:

because the drought stress was introduced primarily before and during the flowering time, is it more precise if we use the term effect of drought stress at reproductive stage? Late-terminal drought seems to give the readers an impression that the drought stress was imposed at grain filling period near harvest.

- The drought experiment in this study starts before heading and at the time of intense stage of drought treatment (56-65 DAS) the genotypes are at the stage of heading or grain filling. However, we did not observe any effect of drought treatment on DHE, but the drought treatment impacted the stages after heading, when plants were in the critical stages of grain filling and seed development. Therefore, we think that the term “late-terminal drought” is appropriate in this context. We added the following sentences to discussion to clarify this point:

The new version reads:

Page 14, lines 404-408: *“…The drought treatment started before start of heading and spanned from 35 DAS to 73 DAS which overlapped with the duration of heading for the population (Figure 2). However, all traits showed decrease under TD except for DHE; possibly due to imposing TD shortly before start of heading. During the intense stage of drought treatment from 56 DAS to 65 DAS, majority of genotypes reached to grain filling and seed development stage (Figure 2), exposing them to late-terminal drought (Samarah et al. 2009; Samarah and Alqudah 2011).…*”

Samarah NH, Alqudah AM, Amayreh JA, McAndrews GM (2009) The effect of late-terminal drought stress on yield components of four barley cultivars. J Agron Crop Sci 195:427–441. https://doi.org/10.1111/j.1439-037X.2009.00387.x

Samarah N, Alqudah A (2011) Effects of late-terminal drought stress on seed germination and vigor of barley (Hordeum vulgare L.). Arch Agron Soil Sci 57:27–32. https://doi.org/10.1080/03650340903191663

Reviewer #2:

Afsharyan et al. title "Effect of developmental genetic mechanisms on grain yield under late-terminal drought reveals favorable QTL alleles in spring barley MAGIC population" conducted QTL mapping to uncover genetic mechanisms grain yield formation under late terminal drought stress in barley. This study conducted phenotyping of yield traits in two years 2011 and 2012 using 534 spring barley MAGIC population. The overall objective of this study is to identify genetic loci associated with grain yield-related traits in response to drought stress. This genetic information is crucial to develop drought tolerant barley cultivars. I found this article is very well-studied, methodology and statistical is sound, results are properly presented. There are few minor corrections need to address before considering its for publication. Some specific comments are below:

- Thank you very much for valuable comments and positive evaluation of our study. The authors appreciate the in-depth description and review of the manuscript. We have carefully considered your comments and have made the necessary revisions to address them to improve the manuscript. The page and line numbers are according to new version of the manuscript.

1. In title "developmental genetic mechanisms" is misleading here. Developmental mechanisms something in-depth pathway-based analysis, while authors only measure some yield attributes. So, title need to modify based on investigated traits and analyses.

- Authors agree that the title can be improved to better represent the analysis and results:

New title reads:

“*Identifying developmental QTL alleles with favorable effect on grain yield components under late-terminal drought in spring barley MAGIC population*”

2. In Figure 1, author only showed soil moisture content. Moreover, it is also crucial to show temperature fluctuation in the same figure for better understanding. During drought stress, temperature also an important factor.

- We already presented supplementary data S1 which includes daily minimum, mean and maximum temperature for growth season in each year. However, we agree that presenting data for daily mean temperature in Figure 1 can help reader better understand the study. Therefore, Figure 1 was updated and daily mean temperature was included for each year:

**Figure 1** Soil moisture content for well-watered (black) and terminal drought (red) treatment as well as daily mean temperature (orange) in 2011 and 2012. The days after sowing (DAS) in the start of reduction in irrigation, permanent wilting point and re-watering is indicated for late-terminal drought treatment.

3. Line 183: please remove treatment from the "well-watered treatment".

- Page 5, line 185: “treatment” was removed.

4. Line 208: please provide details of the sensor.

- Detail of the sensor is provided as follows:

The new version reads:

Page 6, line 209-210: “… *by a HOBO Soil Moisture Smart-Sensor S-SMB-M003 (Onset, MA, USA) in pot …*”

5. Why stress tolerance index (STI) is important to study drought responses, provide few sentences of this importance in introduction.

- The sentences were added to introduction as follows:

The new version reads:

Page 5, line 164-169: “*… In addition to mapping QTL associated with traits under normal and drought conditions, QTL were also detected based on stress indices as phenotype input (Honsdorf et al. 2014; Oyiga et al. 2020). Fernandez et al. (1992) proposed stress tolerance index (STI) which is calculated based on phenotyping data from normal and stress conditions to identify genotypes that show better tolerance under stress condition. This index was used previously to directly detect QTL associated with drought tolerance (Oyiga et al. 2020). …*.”

Fernandez GCJ (1992) STRESS TOLERANCE INDEX-A NEW INDICATOR OF TOLERANCE. HortScience 27:626d—626

Honsdorf N, March TJ, Berger B, et al (2014) High-throughput phenotyping to detect drought tolerance QTL in wild barley introgression lines. PLoS One 9:. https://doi.org/10.1371/journal.pone.0097047

Oyiga BC, Palczak J, Wojciechowski T, et al (2020) Genetic components of root architecture and anatomy adjustments to water-deficit stress in spring barley. Plant Cell Environ 43:692–711. https://doi.org/https://doi.org/10.1111/pce.13683

6. Line 259: only ST is enough as it is abbreviated above.

- Page 8, line 263 and 265: Corrected.

7. In Figure 2, data for DHE is missing.

- Thanks for pointing this out. The data for DHE was added to Figure 2:

**Figure 2** Box-Whisker plots describe the variation for six traits affected by treatment in 534 spring barley MAGIC population under well-watered (WW) and late-terminal drought (TD) treatments using mean values for 2011 and 2012. Trait names and units are indicated above their respective sub-plot. Trait abbreviations are PLH: plant height, AGB: above ground biomass, GFP: grain filling period, NE: number of ears, NK: number of kernels, TKW: thousand kernel weight, DHE: days to heading. Significant treatment effect is indicated with red asterisks with **P* <0.05, ***P* <0.01 and ****P* <0.001. Change in trait mean (%) under terminal drought treatment compared to well-watered is shown below the asterisks.

8. Line 209: Please elaborate BBCH.

- It was elaborated as follows:

The new version reads:

Page 6, line 211-213: “*… when plants were at BBCH 20-32 (beginning of tillering - positioning of node 2 of main stem at least 2 cm above node 1) in 2011 and BBCH 22-30 (detecting 2 tillers - beginning of stem elongation) in 2012…*”

9. In discussion, author need to highlight their own findings then justify with other study.

-We agree with your remark. Even though the discussion has contextualized most of our findings in detail within the broader literature, it can be further improved to ensure that we have adequately emphasized our own findings and their relevance to the previous studies. The following are parts of the discussion that are extended based on your comment

The new version reads:

Page 14, line 414-417: *“… TD most impacted AGB which was positively correlated with PLH (González et al. 1999; Sánchez-Díaz et al. 2002) and NE. Severe impact of drought treatment on aboveground biomass was reported in previous studies (Samarah et al. 2009; Pham et al. 2019)…”*

González A, Martı́n I, Ayerbe L (1999) Barley yield in water-stress conditions.: The influence of precocity, osmotic adjustment and stomatal conductance. F Crop Res 62:23–34

Pham A-T, Maurer A, Pillen K, et al (2019) Genome-wide association of barley plant growth under drought stress using a nested association mapping population. BMC Plant Biol 19:134

Samarah NH, Alqudah AM, Amayreh JA, McAndrews GM (2009) The effect of late-terminal drought stress on yield components of four barley cultivars. J Agron Crop Sci 195:427–441. https://doi.org/10.1111/j.1439-037X.2009.00387.x

Sánchez-Díaz M, García JL, Antolín MC, Araus JL (2002) Effects of soil drought and atmospheric humidity on yield, gas exchange, and stable carbon isotope composition of barley. Photosynthetica 40:415–421

The new version reads:

Page 14, line 431-436: *“…The majority of QTL coincided with known gene/QTL regions with high precision (Supplementary Table S5 - S8). Peak marker BK_12 on chromosome 2H is gene-specific for Ppd-H1 gene (Colmsee et al. 2015) and was detected for traits GFP (WW, TD, M×T) and TKW (TD, ST) which were reported to be associated with Ppd-H1 region (Maurer et al. 2016; Honsdorf et al. 2017). A previous study also reported detecting the same marker for flowering time, which is known to be regulated by Ppd-H1 gene, as an indication of QTL mapping precision in another multi-parental population (Maurer et al. 2015).…”*

Colmsee C, Beier S, Himmelbach A, et al (2015) BARLEX - The barley draft genome explorer. Mol Plant 8:964–966. https://doi.org/10.1016/j.molp.2015.03.009

Honsdorf N, March TJ, Pillen K (2017) QTL controlling grain filling under terminal drought stress in a set of wild barley introgression lines. PLoS One 12:1–18. https://doi.org/10.1371/journal.pone.0185983

Maurer A, Draba V, Jiang Y, et al (2015) Modelling the genetic architecture of flowering time control in barley through nested association mapping. BMC Genomics 16:1–12. https://doi.org/10.1186/s12864-015-1459-7

Maurer A, Draba V, Pillen K (2016) Genomic dissection of plant development and its impact on thousand grain weight in barley through nested association mapping. J Exp Bot 67:2507–2518. https://doi.org/10.1093/jxb/erw070

The new version reads:

Page 15, line 443-446: *“…Vrs1 alleles inherited from six-rowed barley is expected to induce a stronger desirable effect for NK compared to other 2-rowed parental lines and the opposite effect on TKW as higher grain number leads to competition of single grains for assimilates (Maurer et al. 2016)…”*

Maurer A, Draba V, Pillen K (2016) Genomic dissection of plant development and its impact on thousand grain weight in barley through nested association mapping. J Exp Bot 67:2507–2518. https://doi.org/10.1093/jxb/erw070

The new version reads:

Page 16, line 507-509: *“…However, the increase in grain number might have a negative influence on grain weight (Maurer et al. 2016; Calderini et al. 2021) and the impact of the trade-off should be considered on the potential yield.* *…”*

Calderini DF, Castillo FM, Arenas-M A, et al (2021) Overcoming the trade-off between grain weight and number in wheat by the ectopic expression of expansin in developing seeds leads to increased yield potential. New Phytol 230:629–640. https://doi.org/10.1111/nph.17048

Maurer A, Draba V, Pillen K (2016) Genomic dissection of plant development and its impact on thousand grain weight in barley through nested association mapping. J Exp Bot 67:2507–2518. https://doi.org/10.1093/jxb/erw070

10. This study focused only QTL identification. I found some promising QTL associated with terminal drought is very informative. It would be highly recommended if author can further identify causal genes thus regulated flowering time and yield components.

- We appreciate your interest in our research. While we did observe some promising QTL with strong effects on important grain yield components including ones located at known loci such as *Ppd-H1* and *Vrs1*, our study focused solely on QTL identification. The goal of our study was to identify favorable QTL alleles associated with development and grain yield components under late-terminal drought stress in spring barley, and we achieved this goal through various methods of QTL mapping using the MAGIC population. Future studies could investigate the functional mechanisms underlying these QTL by identifying the causal genes and exploring their effects on flowering time and yield components. Such work would be a valuable avenue for future research in this area and could provide a deeper understanding of the genetic mechanisms underlying yield responses to drought stress in spring barley. However, such a study would require a more extensive set of experiments and data analysis, including transcriptomics, proteomics, and metabolomics which is out of the scope of the current study.
